# Case Report: Metagenomic Next-Generation Sequencing Can Contribute to the Diagnosis and Treatment of Disseminated Visceral Kaposi Sarcoma Following Allogeneic Haematopoietic Stem Cell Transplantation

**DOI:** 10.3389/fonc.2022.848976

**Published:** 2022-02-24

**Authors:** Kuangguo Zhou, Jinhuan Xu, Zhen Shang, Hanying Sun, Kefeng Shen, Yi Xiao

**Affiliations:** Department of Hematology, Tongji Hospital, Tongji Medical College, Huazhong University of Science and Technology, Wuhan, China

**Keywords:** haematopoietic stem cell transplantation, kaposi sarcoma, human herpesvirus 8, metagenomic next-generation sequencing, immunosuppression, genetic predisposition

## Abstract

Disseminated visceral Kaposi sarcoma (KS) following allogeneic haematopoietic stem cell transplantation (HSCT) is a rare but life-threatening posttransplant complication. A suitable management strategy for disseminated KS involvement in transplant patients is unclear. Here, we reported a patient who developed disseminated visceral KS following HSCT, which was the first detailed report documenting the relationship among KS development, delayed immune reconstitution, and HHV-8 DNA levels by metagenomic next-generation sequencing (mNGS). The HHV-8 viral load peaked at 2071 sequence reads with an absolute lymphocyte count of 0.17×10^9^/L on day +242. On day +536, the HHV-8 viral load became undetectable, with an absolute lymphocyte count of 1.06×10^9^/L and the KS disappearance. HHV-8 load in blood detected by mNGS may be used as an early prediction marker for KS, a guide for early withdrawal of immunosuppression, and a tool to monitor KS treatment response in the setting of HSCT, especially in patients with CMV-seropositive or graft failure postengraftment. Through whole-exome sequencing, we explored the molecular mechanism underlying the patient’s longer latency of haematopoietic or immune reconstitution and recurrent infections. Germline mutations in the FANCI and RAD51 genes might impair the patient’s DNA repair ability, leading to a degree of immunodeficiency and tumour susceptibility. We strongly recommended evaluating the clinical history of the donor and investigating whether there were possible germline mutations suspected for immunodeficiency or familial neoplasms. Disseminated visceral KS patients could likely benefit from chemotherapy, especially if the disease appears to be aggressive.

## Introduction

Kaposi sarcoma (KS) is a vascular tumour characterized by abnormal angioproliferation by immunosuppression and was first described by Moritz Kaposi in 1872. Gamma herpesvirus human herpes virus 8 (HHV-8), also known as Kaposi sarcoma-associated herpes virus (KSHV), was subsequently found to be associated with the development of KS in 1994 (1). Later, HHV-8 was recognized to be linked with other lymphoproliferative diseases, such as multicentric Castleman disease and primary effusion lymphoma, and to be a cause of bone marrow suppression (2). HHV-8 could become latent due to its complex interference with the immune system. HHV-8 seroprevalence varies worldwide, with 2-4% in Asia and Northern Europe and 40% in Saharan Africa. Approximately 5-20% of patients in the United States and 10% of patients in Mediterranean countries are seropositive for HHV-8. On the basis of the prevalence of HHV-8 infection, the incidence of KS ranges from 0.6 to 5.3% (3). Clinical manifestations of KS include mucocutaneous and visceral involvement. Disseminated disease with visceral involvement is unusual and has a poor prognosis.

Unlike the classic KS form, posttransplant KS (PT-KS) is a rare but often life-threatening posttransplant complication characterized by a rapidly progressive course. The treatment of KS in transplant recipients is particularly complicated because immunosuppression reduces the risk of graft rejection, and when immunosuppressed patients receive chemotherapy, the risk of infection increases. Although PT-KS often occurs after solid organ transplantation (SOT, especially kidney transplantation, 1-4.1%) (4), there are few reports of KS after haematopoietic stem cell transplantation (HSCT). The mechanism has been proposed to be multifactorial. First, SOT patients were usually linked with longer course of immunosuppression treatment. Second, the intense cytotoxic conditioning regimens in HSCT destroyed the host lymphoid systems and latent HHV-8-harbouring cells, while the immunosuppressive regimens used in SOT did not eradicate such cells. Impaired T cell immunity was considered to be related to KS development. Due to the increase in human leukocyte antigen (HLA)-mismatched donor allogeneic HSCT (alloHSCT) and anti-thymocyte globulin (ATG) for graft-versus-host disease (GVHD) prevention (5), the frequency of KS after HSCT has increased gradually (1, 5–7). In most reported cases, the patient died in less than 1 year during the follow-up period. However, little is known about the potential association of lymphocyte number with circulating HHV-8 DNA levels during different periods after HSCT (8, 9). Trials in this field have not been feasible due to the rarity of disease. The guidelines for optimal management of disseminated KS in transplant patients are not clear, and the prognosis are inferior.

HHV-8 virus loads in blood have been considered to be a predictor of the development of clinical KS. Currently, since there are no validated commercial serology kits, the testing of donors and recipients for HHV-8 is challenging (10). Since its emergence in 2004, the cost of high-throughput or next-generation sequencing (NGS) has been reduced by several orders of magnitude (11, 12). As an emerging method of pathogen detection, metagenomic next-generation sequencing (mNGS) is an innovative technological platform that has the promise of improving our ability to diagnose and track infectious diseases. HHV-8 load detected by mNGS may be used as an early prediction marker for PT-KS. Additionally, whole-exome sequencing (WES) has vastly improved the understanding of the molecular mechanism of haematological malignancies and immunodeficiency disease (13, 14). Based on the WES platform, in this case, we explored the patient’s germline genetic predisposition for the development of significantly delayed haematopoietic immune reconstitution after transplantation. Written informed consent was obtained from the patient for the publication of any potentially identifiable images or data included in this article.

This case illustrated the rise and fall of disseminated visceral Kaposi sarcoma coinciding with immunosuppression and reconstitution after HSCT in a 29-year-old Asian patient. Here, we propose that more importance should be attached to the early diagnosis and better understanding of KS after transplantation, especially in recipients who have HHV-8 risk factors such as HLA-mismatched donor allogeneic transplants or those who receive ATG for GVHD prevention. Moreover, our study suggests that routinely performed germline analysis of immunodeficiency-associated mutations might contribute to optimizing alloHSCT from a related donor to avoid delayed haematopoietic or immune reconstitution and disease recurrence after transplantation.

## Case Presentation

A 29-year-old Chinese male patient was admitted to Tongji Hospital in August 2019 due to “skin ecchymosis with low fever for 2 weeks”. The patient was diagnosed with acute myeloid leukaemia AML-M2, t (8, 21) (q22; q11), -Y, AML1/ETO (+), c-Kit D816Y mutation, indicating a high risk of disease relapse (15, 16). After standard “3+7” induction therapy and two consolidation regimens included high dose cytarabine, his bone marrow had morphological remission. However, the level of AML1/ETO fusion genes (1.57% after three chemotherapy courses) declined less than 3 logarithms compared with that at the preliminary diagnosis (84.67% before leukaemia treatment), which predicted a poor prognosis (17, 18). As the patient consistently showed positive minimal residual disease (MRD), HSCT was recommended after consent was received from the patient and his family (19). Both the patient and his 51-year-old father as donor (haploidentical HLA-matched, 5/10, with no history of neoplasia or immunodeficiency disease) were serum-negative for cytomegalovirus (CMV, also called HHV-5), Epstein–Barr virus (EBV, also called HHV-4), hepatitis B and C viruses, and human immunodeficiency virus (HIV).

**Figure 1:**
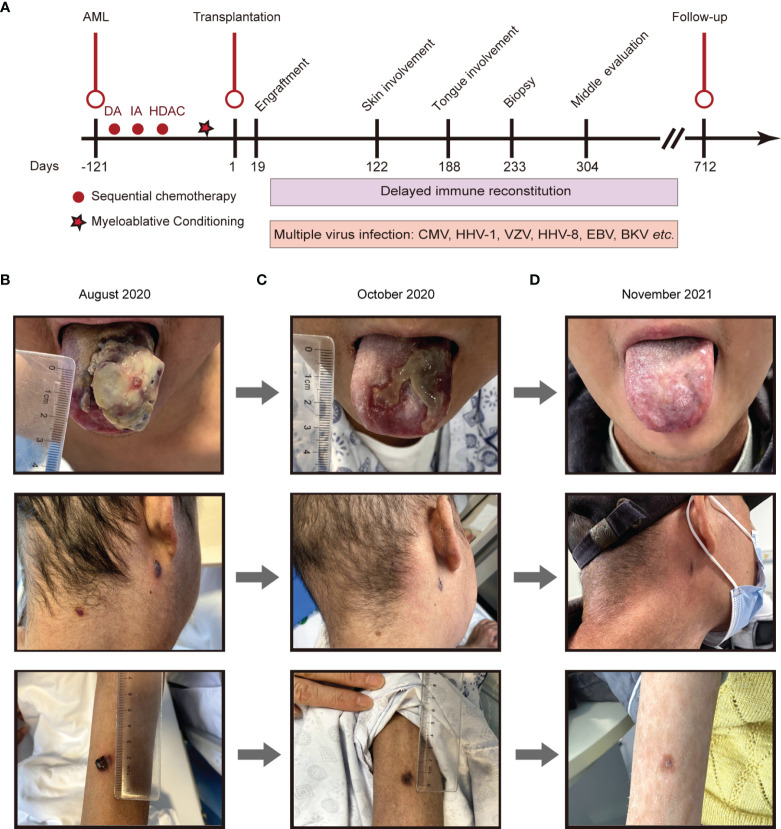
The disease development of the patient from different periods. **(A)**, Main timeline of patient disease development. **(B-D)**, Different periods of Kaposi sarcoma on the tongue, cervical skin, and skin of the right forearm. **(B)**, August 13th, 2020; **(C)**, October 14th, 2020; **(D)**, November 26th, 2021. DA, daunorubicin hydrochloride and cytarabine; IA, idarubicin hydrochloride and cytarabine; HDAC, high dose cytarabine.

In December 2019, the patient underwent allogeneic haploidentical HSCT using a standard dose of the Bu/Cy/AraC/MeCCNU/ATG conditioning regimen (20). The patient received bone marrow blood (0.43×10^6^/kg CD34^+^ cells) and peripheral blood mononuclear cells (1.32× 10^6^/kg CD34^+^ cells) on day +1 (**Figure 1A**). All blood products, including packed cells and platelets, were irradiated with gamma rays for posttransfusion GVHD prevention. Granulocyte engraftment occurred on day +16, and platelets were engrafted on day +19. In the early posttransplant period, the patient developed severe oral mucositis (Grade IV) on day +12 in World Health Organization (WHO) scale (21), acute skin GVHD on day +17 (Grade II) (22) and a persistent fever on day +11. Traditional pathogen identification tests were negative, such as blood culture for bacteria or fungi and reverse-transcription PCR (RT-PCR) of multiple viruses, except for EBV (3.7×10^4^copies/mL by RT-PCR). As an emerging method of pathogen detection, mNGS in blood on day +13 showed that he was also infected with cytomegalovirus (CMV, 426 sequence reads) and herpes simplex virus (HHV-1, 1275 sequence reads). Antiviral therapy with ganciclovir was promptly instituted. When he was discharged on day +30, blood tests showed almost normal counts of white blood cells (8.07×10^9^/L), neutrophils (6.13×10^9^/L), lymphocytes (1.08×10^9^/L), and platelets (214×10^9^/L), and haemoglobin levels (108 g/L). Bone marrow biopsy showed normalization of trilineage haematopoiesis and complete donor engraftment of 97.6% by DNA-STR amplification. After discharge, the patient was treated with the immunosuppressants cyclosporine and prednisone for GVHD prevention, antiviral drugs ganciclovir, antifungal prophylaxis (voriconazole), and anti-pneumocystis prophylaxis (sulfamethoxazole), but his oral mucositis persisted (Grade II).

**Figure 2:**
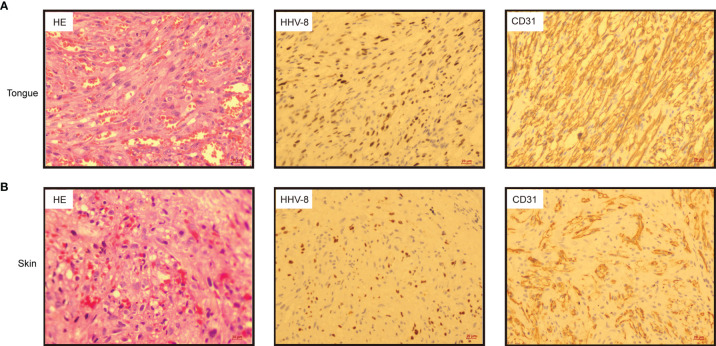
Histologic sections from tongue **(A)** and skin of the right arm **(B)** exhibited spindle cell proliferation in bundles, extravasated red blood cells and hyaline globules. (Left panel, haematoxylin & eosin, HE 20×); Immunohistochemistry indicated that the tumour cells were positive for human herpesvirus 8 (HHV-8) (middle panel, HHV-8 20×) and CD31 (right panel, CD31 20×).

On day +93, the patient developed herpes zoster affecting the right chest and waist with anti-varicella-zoster virus (VZV, HHV-3) IgM positive in local hospital, which was alleviated by foscarnet sodium for two weeks and topical medication. On day +122, the patient experienced intermittent epigastric discomfort and progressive emaciation, and multiple dark red or purplish small nodules appeared on his extremities and neck without pain or itch; however, he did not take it seriously, and no special treatment was given. On day +188, the patient’s tongue was scratched by a fishbone when he ate fish. A lingual mass gradually appeared, which affected his eating. On day +218, he was admitted again complaining of dysphagia and polypnea and was unable to eat due to the painful, rapidly growing tongue mass. Physical examination showed an exogenous and lobulated mass (about 4.5 cm × 3.5 cm) on the back of his tongue, with a clear boundary and medium texture (**Figure 1B**). In addition, masses were palpable in the posterior area of the right molar, the right upper palate and the left buccal mucosa, with a clear boundary and medium texture. Multiple skin nodules (greater than ten) were found on the extremities accompanied by chronic skin GVHD lesions such as depigmentation and papulosquamous lesions. Biopsies obtained from the tongue mass on day +233 (**Figure 2A**) and skin nodule of the right arm (**Figure 2B**) showed variable degrees of spindle cell proliferation and were positive for HHV-8, confirming a diagnosis of Kaposi sarcoma. The immunohistochemical results were HHV-8+, CD34+, CD31+, ERG+, FLI1+, PCK-, EMA-, and Ki67 30%. PET-CT showed disseminated tumour involvement in his skin, mouth, tongue, lung, oesophagus, and gastrointestinal tract. mNGS in blood verified HHV-8 infection with 2071 sequence reads. However, RT-PCR showed that the HHV-8 viral load was negative. Blood tests showed pancytopaenia, white blood cells 1.52×10^9^/L, neutrophils 1.21×10^9^/L, lymphocytes 0.17×10^9^/L, haemoglobin 70 g/L, and platelets 32×10^9^/L. Liver and kidney functions were normal with hypoproteinaemia. Bone marrow (BM) biopsy displayed that marrow hypoplasia and continued leukaemia remission with complete donor engraftment (**Table 1**).

**Table 1:** Clinical features and HHV-8 virologic study of the patient after allogeneic haematopoietic stem cell transplantation.

	August 13^th^, 2020	October 14^th^, 2020	November 26^th^, 2021
White blood cell (×10^9^/L)	1.52	2.59	4.91
Absolute lymphocyte count (×10^9^/L)	0.17	0.27	0.92
CD3+CD19- T lymphocytes/µl	99	76	394
CD3^+^CD4^+^ T lymphocytes/µl	44	30	122
CD3^+^CD8^+^ T lymphocytes/µl	54	45	263
CD3-CD19+ B lymphocytes/µl	10	4	14
CD3-/CD16+CD56+ NK lymphocytes/µl	50	79	788
Neutrophil (×10^9^/L)	1.21	2.2	2.92
Haemoglobin (g/L)	77	61	111
Platelet (×10^9^/L)	29	28	144
IgA (g/L)	0.59	NA	0.74
IgG (g/L)	5	NA	11
IgM (g/L)	0.33	NA	0.33
HHV-8 detection sequence reads (Total sequencing data)	2071 (296.7 M)	10 (336 M)	0 (362 M)
AML1/ETO	0	0	0
C-Kit mutation	0	0	0
Chimeric rate (T lymphocytes)	99.50%	NA	99.82%
Chimeric rate (Bone marrow)	100%	NA	99.4%

NA, Not Available.

Given the urgency and complexity of simultaneously controlling the patient’s progressive disseminated KS and his persistent GVHD, the patient’s treatment was adjusted by converting the immunosuppressant cyclosporine to sirolimus (23) and using liposomal doxorubicin (20 mg q3w for only 3 times, limited by severe pancytopenia) (24), thalidomide (75 mg qn) (25), antiviral drugs (foscarnet sodium, ganciclovir was limited by severe pancytopenia), topical alitretinoin (25), and 5% topical imiquimod ointment. After two months, the recipient responded well to the treatment described above (**Figure 1C**). Multiple KS lesions in the patient’s skin, oral cavity, and tongue gradually improved significantly as the HHV-8-DNA level decreased sharply, as detected by mNGS. On day +275, the patient was diagnosed with BK virus (BKV)-related haemorrhagic cystitis (1.33×10^5^ copies/ml in urine, grade III) with frequent urination with haematuria. After hydration, alkalization, and antiviral (foscarnet sodium) treatment, his haemorrhagic cystitis was ameliorated. However, on day +326, the patient suffered from severe pneumonia with hypoxemia and secondary sepsis. During this period, the patient’s BM biopsy consistently showed panhypocellularity along with severe pancytopaenia. Until approximately 430 days after alloHSCT, his complete blood count gradually recovered.

**Figure 3:**
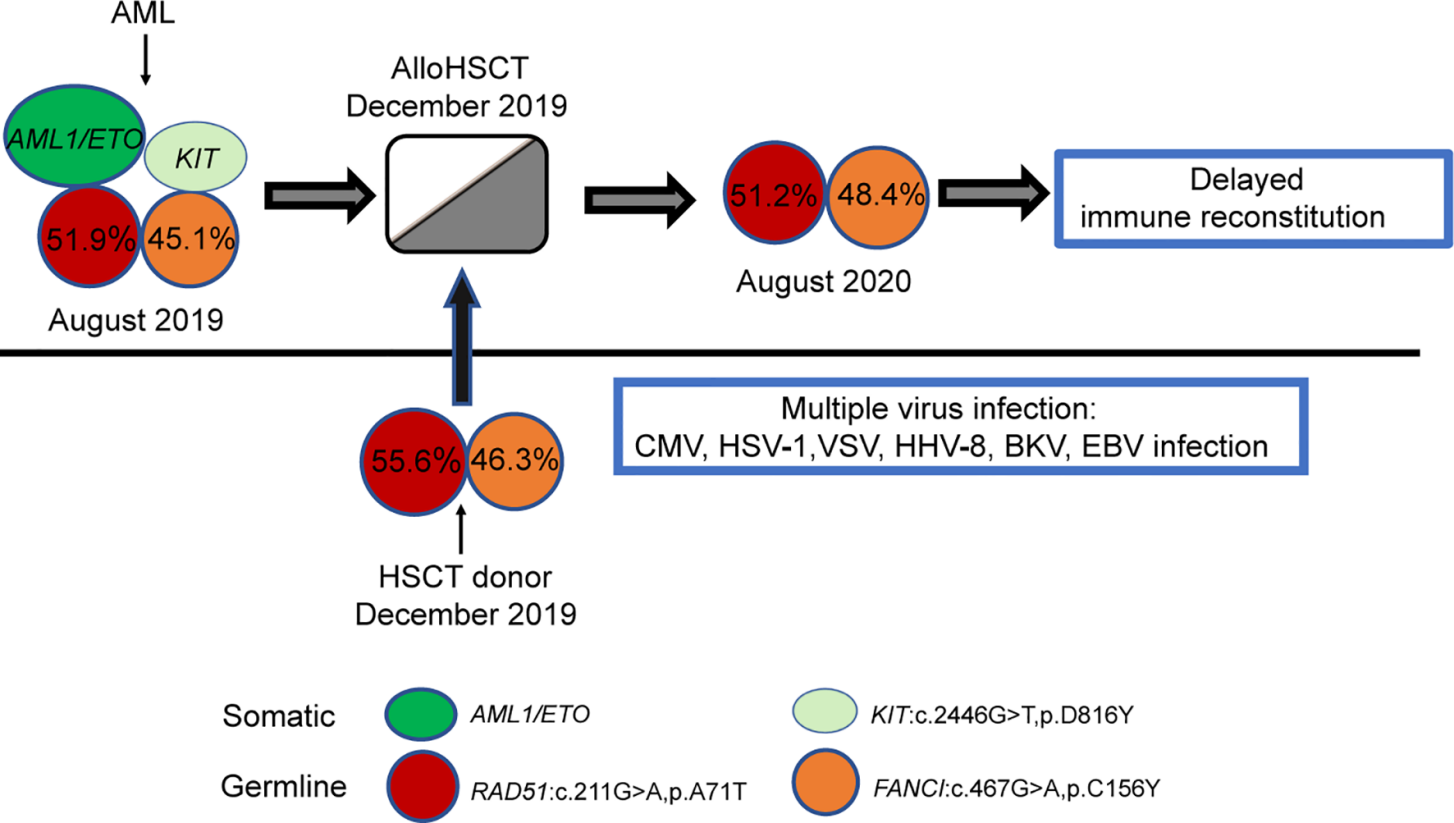
Model depicting the role of germline mutations in the FANCI and RAD51 genes in the case. Each coloured circle represents a specific mutation, and the numbers depicted show the variant allele frequency. Variant allele frequency was obtained through the proportion of variant reads for a particular sequence.

To determine why the patient’s haematopoietic or immune reconstitution was delayed, WES was performed on the patient’s samples before and after transplantation. Two germline mutations related to delayed immune reconstitution (FANCI: c.467G>A, p. C156Y, RAD51: c.211G>A, p. A71T) were detected (**Figure 3**). Interestingly, the same mutations were observed in his father, which proved their germline origin.

At the last follow-up (+712 days), the patient still received immunosuppressive therapy with low-dose ruxolitinib (5 mg per 3 days) and thalidomide (75 mg qn) to treat mild cutaneous chronic skin rejection (Grade II) (22, 26). He remained well with complete donor chimaerism and in complete leukaemia remission. Consistent with the absence of HHV-8 infection in blood as detected by mNGS, neither Kaposi sarcoma nor lymphoproliferative disease recurred after the follow-up period of over two years (**Figure 1D**). Through mNGS, we found that as the lymphocytes recovered, the HHV-8 load gradually decreased, consistent with KS disappearance (**Table 1**). In parallel, the patient’s father had been regularly evaluated, and his peripheral blood counts and immunity remained normal.

## Discussion

HHV-8 infection is quite common after HSCT. However, PT-KS, particularly its disseminated visceral form, is a very rare complication, and its relationship with HHV-8 has been reported infrequently (27, 28). In this study, we reported a patient who developed disseminated visceral KS following HSCT, which predicted an inferior prognosis. Based on our comprehensive literature review, this is the first detailed report to document the relationship between KS development and delayed immune reconstitution with HHV-8 levels detected by mNGS in blood. An increase in HLA-mismatched donor allogeneic transplants and the use of ATG for GVHD prevention have been proposed as factors that predispose recipients to KS. The suitable management of disseminated KS involvement in transplant patients is not clear.

The presence of HHV-8 has been described as an obligatory diagnostic criterion for KS. Given the variable sensitivity and specificity and lack of standardization, HHV-8 serologic detection was not usually included in pretransplant screening. After HSCT, HHV-8 antibody responses may be damaged owing to immunosuppression. The traditional method RT-PCR has limited sensitivity. Our study indicated that mNGS in blood could provide fast and precise pathogen detection, contributing to the assessment of KS development and guiding treatment decisions dynamically. The HHV-8 viral load peaked at 2071 sequence reads with an absolute lymphocyte count of 0.17×10^9^/L on day +242. On day +304, there was a remarkable decrease in skin and tongue lesions coinciding with HHV-8 viral load decreasing to 10 sequence reads. On day +536, the HHV-8 viral load became undetectable, with an absolute lymphocyte count of 1.06×10^9^/L and KS disappearance. HHV-8 infection may be spread through a donor’s latent infection, blood transfusions, or reactivation of the recipient’s previous infection. HHV-8 was found to be negative by mNGS in his haploidentical HLA-matched donor. The origin of HHV-8 infection in our case was unclear since no test was performed on the recipient prior to HSCT. Hence, the screening of donors should be improved to prevent infection with HIV, HHV-8, CMV, and other viruses through transplantation.

The therapeutic cornerstone in the posttransplant KS management was based on 3 pillars: immunosuppression reduction, conversion to mammalian target of rapamycin inhibitors, and chemotherapy (7, 29). Tapering down immunosuppressive regimens to the lowest possible level is the main treatment for PT-KS, but it is limited by the risk of graft rejection. Calcineurin inhibitors such as cyclosporine and tacrolimus have been suggested to promote KS progression *via* vascular endothelial growth factor (VEGF) upregulation. On the contrary, Lupinacci and Delyon et al. reported that mammalian target of rapamycin inhibitors, such as sirolimus, could treat KS in renal transplant recipients based on the capacity of sirolimus to abrogate VEGF-induced tumour proliferation (4, 23). Therefore, we adjusted the immunosuppressant (cyclosporine converted to sirolimus). Although there was no consensus on chemotherapy for posttransplant KS, several acquired immune deficiency syndrome (AIDS)-related KS studies revealed that patients with diffuse involvement could benefit from systemic chemotherapy (30, 31). Based on the evidence of disseminated KS in our patient, pegylated liposomal doxorubicin, which was effective in treating diffuse AIDS-related KS, was initiated. Hence, for patients with disseminated visceral involvement, we suggest initial systemic chemotherapy with liposomal anthracycline might be appropriate.

Viral infections had become a major challenge for stem cell transplant recipients, resulting in a high mortality. In our study, HHV-1/VZV (HHV-3)/EBV (HHV-4)/CMV (HHV-5)/KSHV (HHV-8)/BKV infection occurred successively in the patient, which has not been reported previously. Mixed infections of two or three herpesviruses, mostly CMV and EBV infections, have been documented, and other herpesviruses include successive CMV/HHV-8, HHV-6/CMV, and EBV/HHV-7 infections. Cesaro et al. suggested that CMV infection of previously HHV-8-infected human fibroblasts could reactivate the viral lytic cycle of HHV-8 *in vitro* and then HHV-8 viremia and KS (1). Viral infection after transplantation had a linkage and waterfall effect. We should closely monitor for infections such as CMV or HHV-8. mNGS showed promising potential in pathogenic diagnosis during posttransplant infections and might help physicians make targeted therapeutic decisions timely. Additionally, strategies to carry out early and thorough antiviral treatment are urgently needed.

The recurrent HHV-1/CMV/EBV/VZV/HHV-8/BKV infections strongly supported the hypothesis of impaired immunosurveillance in this case. To investigate the mechanism underlying the longer latency of haematopoietic or immune reconstitution, we also performed WES on the patient and his father. Two germline mutations (FANCI: c.467G>A, p. C156Y, and RAD51: c.211G>A, p. A71T) were detected. FANCI is a well-known pathogenic gene for the Fanconi Anaemia (FA), a progressive bone marrow failure syndrome that affects DNA repair process and increases cancer risk, including AML. RAD51 is a newly identified gene also contributing to FA. Interestingly, the patient in our study had no clinical symptoms that pointed to FA, such as microcephaly, abnormal thumbs, slow growth rate before leukaemia onset. However, germline mutations in the FANCI and RAD51 genes might impair the DNA repair ability, lead to a certain degree of immunodeficiency and tumour susceptibility of the patients, and thereby make the patients sensitive to pathogen infection, ionizing radiation, and other damages (32). There was a close relationship between FA related genes and immune deficiency. The International Federation of immune Societies had also included more than 20 FA genes in the catalogue of pathogenic genes of congenital immune deficiency (33). Hence, we speculated on the potential pathogenesis of the patient during the different periods of disease development. The patient was born with FANCI gene C156Y and RAD51 gene A71T germline mutations, both of which were inherited from his father. Over time, some of the patient’s haematopoietic stem progenitor cells underwent somatic mutations (developing a c-Kit gene D816Y mutation and an AML1/ETO fusion), which led to AML. As the patients showed consistently positive MRD based on AML1/ETO testing, the patient underwent haploidentical HSCT with his father as the donor. The donor-derived haematopoietic stem cells carried germline mutations of the FANCI and RAD51 genes and were functionally “congenitally insufficient”. Haematopoietic immune reconstruction was significantly delayed after implantation in the recipient. During the posttransplant period, multiple viruses’ infections occurred successively. Therefore, our study suggested that routinely performed germline analysis of immunodeficiency-associated mutations might contribute to optimizing alloHSCT from a related donor to avoid delayed haematopoietic or immune reconstitution and disease recurrence after transplantation. All potential related donors, especially in the family with immunodeficiency or tumour history, should be screened for the FA-related mutations before HSCT to avoid failed engraftment, impaired immune reconstitution post-HSCT, or donor-derived tumours.

Although KS is a rare complication of HSCT, it should be given more attention, particularly to recipients of haploidentical HLA-matched alloHSCT who had more intensive immunosuppressive therapy or used ATG to prevent GVHD. HHV-8 DNA detection in blood by mNGS may be used as an early identification marker for KS in the setting of HSCT, especially in patients with CMV seropositivity or graft failure post-engraftment, and may be used as a guide for early withdrawal of immunosuppression and a tool to monitor KS treatment response. This study has a few limitations. This is a retrospective case report that lacks basic research. Based on this single case, it is difficult to draw a strong conclusion. Further research is needed to standardize the treatment for HHV-8-associated KS after transplantation. Evaluation of the clinical history of the donor and investigation of whether there are possible germline mutations suspicious for familial neoplasms are strongly recommended.

## Data Availability Statement

The raw data supporting the conclusions of this article will be made available by the authors, without undue reservation.

## Ethics Statement 

Ethical review and approval was not required for the study on human participants in accordance with the local legislation and institutional requirements. The patients/participants provided their written informed consent to participate in this study. Written informed consent was obtained from the individual(s) for the publication of any potentially identifiable images or data included in this article.

## Author Contributions

Conception and design: KZ, YX. Acquisition of data: KZ, JX, ZS, HS, KS, YX. Analysis and interpretation of data: KZ, KS, YX. Writing, review and/or revision of the manuscript: KZ, KS, YX. Study supervision: KZ, YX. All authors contributed to the article and approved the submitted version.

## Funding

This work was supported by the Natural Science Foundation of China [no. 81873444& 82070213] and the Key Research and Development Program of Hubei Science and Technology Department [2020BCB021].

## Conflict of Interest

The authors declare that the research was conducted in the absence of any commercial or financial relationships that could be construed as a potential conflict of interest.

## Publisher’s Note

All claims expressed in this article are solely those of the authors and do not necessarily represent those of their affiliated organizations, or those of the publisher, the editors and the reviewers. Any product that may be evaluated in this article, or claim that may be made by its manufacturer, is not guaranteed or endorsed by the publisher.

## References

[1] Cesaro S Tridello G van der Werf S Bader P Socie G Ljungman P. Incidence and Outcome of Kaposi Sarcoma After Hematopoietic Stem Cell Transplantation: A Retrospective Analysis and a Review of the Literature, on Behalf of Infectious Diseases Working Party of EBMT. Bone Marrow Transplant (2020) 55(1):110–6. doi: 10.1038/s41409-019-0644-8 31435035

[2] Naimo E Zischke J Schulz TF. Recent Advances in Developing Treatments of Kaposi's Sarcoma Herpesvirus-Related Diseases. Viruses-Basel (2021) 13(9):1797. doi: 10.3390/v13091797 PMC8473310 34578378

[3] Deauna-Limayo D Rajabi B Qiu W Htut M Sweetenham J. Kaposi Sarcoma After non-Myeloablative Hematopoietic Stem Cell Transplant: Response to Withdrawal of Immunosuppressant Therapy Correlated With Whole Blood Human Herpesvirus-8 Reverse Transcriptase-Polymerase Chain Reaction Levels. Leuk Lymphoma (2013) 54(10):2299–302. doi: 10.3109/10428194.2013.769221 23343181

[4] Delyon J Rabate C Euvrard S Harwood CA Proby C Gulec AT. Management of Kaposi Sarcoma After Solid Organ Transplantation: A European Retrospective Study. J Am Acad Dermatol (2019) 81(2):448–55. doi: 10.1016/j.jaad.2019.03.028 30902727

[5] Lu DP Dong LJ Wu T Huang XJ Zhang MJ Han W. Conditioning Including Antithymocyte Globulin Followed by Unmanipulated HLA-Mismatched/Haploidentical Blood and Marrow Transplantation can Achieve Comparable Outcomes With HLA-Identical Sibling Transplantation. Blood (2006) 107(8):3065–73. doi: 10.1182/blood-2005-05-2146 16380454

[6] Bruno B Sorasio R Barozzi P Vieira J Omede P Giaretta F. Kaposi's Sarcoma Triggered by Endogenous HHV-8 Reactivation After non-Myeloablative Allogeneic Haematopoietic Transplantation. Eur J Haematol (2006) 76(4):342–7. doi: 10.1111/j.1600-0609.2005.00601.x 16519707

[7] Venkateswaran N Ramos JC Cohen AK Alvarez OP Cohen NK Galor A. Spotlight on Ocular Kaposi's Sarcoma: An Update on the Presentation, Diagnosis, and Management Options. Expert Rev Ophthalmol (2021) 16(6):477–89. doi: 10.1080/17469899.2021.1962294 PMC9624465 36325272

[8] Heyrman B De Becker A Schots R. A Case Report of Immunosuppression-Related Kaposi's Sarcoma After Autologous Stem Cell Transplantation. BMC Res Notes (2016) 9:188. doi: 10.1186/s13104-016-1991-9 27012530 PMC4806455

[9] Hogan LE Hanhauser E Hobbs KS Palmer CD Robles Y Jost S. Human Herpes Virus 8 in HIV-1 Infected Individuals Receiving Cancer Chemotherapy and Stem Cell Transplantation. PloS One (2018) 13(5):e0197298. doi: 10.1371/journal.pone.0197298 29746555 PMC5944966

[10] Dollard SC Annambhotla P Wong P Meneses K Amin MM La Hoz RM. Donor-Derived Human Herpesvirus 8 and Development of Kaposi Sarcoma Among 6 Recipients of Organs From Donors With High-Risk Sexual and Substance Use Behavior. Am J Transplant (2021) 21(2):681–8. doi: 10.1111/ajt.16181 PMC7891580 32633035

[11] Gu W Miller S Chiu CY. Clinical Metagenomic Next-Generation Sequencing for Pathogen Detection. Annu Rev Pathol (2019) 14:319–38. doi: 10.1146/annurev-pathmechdis-012418-012751 PMC6345613 30355154

[12] Wang C Li A Shi Q Yu Z. Metagenomic Next-Generation Sequencing Clinches Diagnosis of Leishmaniasis. Lancet (2021) 397(10280):1213. doi: 10.1016/s0140-6736(21)00352-4 33773632

[13] Farina M Bernardi S Gandolfi L Zanaglio C Morello E Turra A. Case Report: Late Onset of Myelodysplastic Syndrome From Donor Progenitor Cells After Allogeneic Stem Cell Transplantation. Which Lessons Can We Draw From the Reported Case? Front Oncol (2020) 10:564521. doi: 10.3389/fonc.2020.564521 33178592 PMC7591784

[14] Godley LA Shimamura A. Genetic Predisposition to Hematologic Malignancies: Management and Surveillance. Blood (2017) 130(4):424–32. doi: 10.1182/blood-2017-02-735290 PMC5533201 28600339

[15] Paschka P Marcucci G Ruppert AS Mrózek K Chen H Kittles RA. Adverse Prognostic Significance of KIT Mutations in Adult Acute Myeloid Leukemia With Inv(16) and T (8,21): A Cancer and Leukemia Group B Study. J Clin Oncol (2006) 24(24):3904–11. doi: 10.1200/jco.2006.06.9500 16921041

[16] Cairoli R Beghini A Grillo G Nadali G Elice F Ripamonti CB. Prognostic Impact of C-KIT Mutations in Core Binding Factor Leukemias: An Italian Retrospective Study. Blood (2006) 107(9):3463–8. doi: 10.1182/blood-2005-09-3640 16384925

[17] Hu GH Cheng YF Lu AD Wang Y Zuo YX Yan CH. Allogeneic Hematopoietic Stem Cell Transplantation can Improve the Prognosis of High-Risk Pediatric T (8,21) Acute Myeloid Leukemia in First Remission Based on MRD-Guided Treatment. BMC Cancer (2020) 20(1):553. doi: 10.1186/s12885-020-07043-5 32539815 PMC7294617

[18] Jourdan E Boissel N Chevret S Delabesse E Renneville A Cornillet P. Prospective Evaluation of Gene Mutations and Minimal Residual Disease in Patients With Core Binding Factor Acute Myeloid Leukemia. Blood (2013) 121(12):2213–23. doi: 10.1182/blood-2012-10-462879 23321257

[19] Pollyea DA Bixby D Perl A Bhatt VR Altman JK Appelbaum FR. NCCN Guidelines Insights: Acute Myeloid Leukemia, Version 2.2021. J Natl Compr Canc Netw (2021) 19(1):16–27. doi: 10.6004/jnccn.2021.0002 33406488

[20] Koh LP Rizzieri DA Chao NJ. Allogeneic Hematopoietic Stem Cell Transplant Using Mismatched/Haploidentical Donors. Biol Blood Marrow Transplant (2007) 13(11):1249–67. doi: 10.1016/j.bbmt.2007.08.003 17950913

[21] Lalla RV Sonis ST Peterson DE. Management of Oral Mucositis in Patients Who Have Cancer. Dent Clin North Am (2008) 52(1):61–77, viii. doi: 10.1016/j.cden.2007.10.002 18154865 PMC2266835

[22] Martino R Romero P Subira M Bellido M Altes A Sureda A. Comparison of the Classic Glucksberg Criteria and the IBMTR Severity Index for Grading Acute Graft-Versus-Host Disease Following HLA-Identical Sibling Stem Cell Transplantation. Bone Marrow Transplant (1999) 24(3):283–7. doi: 10.1038/sj.bmt.1701899 10455367

[23] Lupinacci S Perri A Toteda G Vizza D Lofaro D Pontrelli P. Rapamycin Promotes Autophagy Cell Death of Kaposi's Sarcoma Cells Through P75NTR Activation. Exp Dermatol (2021) 111. doi: 10.1111/exd.14438 34331820

[24] Dalu D Fasola C Ammoni L De Francesco D Cona MS Rota S. Pegylated Liposomal Doxorubicin as First Line Treatment in Aids-Related Kaposi's Sarcoma: A Real-Life Study. J Chemother (2021) 33(5):342–7. doi: 10.1080/1120009x.2021.1920248 34060438

[25] de Medeiros BC Rezuke WN Ricci A Jr. Tsongalis G Shen PUF Bona RD. Kaposi’s Sarcoma Following Allogeneic Hematopoietic Stem Cell Transplantation for Chronic Myelogenous Leukemia. Acta Haematol (2000) 104(2-3):115–8. doi: 10.1159/000039743 11154986

[26] Zeiser R Polverelli N Ram R Hashmi SK Chakraverty R Middeke JM. Ruxolitinib for Glucocorticoid-Refractory Chronic Graft-Versus-Host Disease. N Engl J Med (2021) 385(3):228–38. doi: 10.1056/NEJMoa2033122 34260836

[27] Anderson MA Ying T Wyburn K Ferguson PM Strach MC Grimison P. Transplant-Associated Penile Kaposi Sarcoma Managed With Single Agent Paclitaxel Chemotherapy: A Case Report. BMC Urol (2021) 21(1):87. doi: 10.1186/s12894-021-00855-y 34098936 PMC8186205

[28] Valero-Arrese L Benitez-Carabante MI Soques Vallejo E Roca I Navarro Jimenez A Diaz-de-Heredia C. Kaposi Sarcoma in a Child After Hematopoietic Stem Cell Transplantation: Should Pre-Transplant HHV-8 Screening be Considered in Recipients From High Prevalence Areas? Transpl Infect Dis (2021) 23(3):e13525. doi: 10.1111/tid.13525 33231901

[29] Bekoz H Sağırlı A Türk O Ceylan B Sargın D. A Rare Complication of Hematopoietic Stem Cell Transplantation: Kaposi Sarcoma. Leukemia Res (2019) 85:S53. doi: 10.1016/s0145-2126(19)30332-7

[30] Cianfrocca M Lee S Von Roenn J Tulpule A Dezube BJ Aboulafia DM. Randomized Trial of Paclitaxel Versus Pegylated Liposomal Doxorubicin for Advanced Human Immunodeficiency Virus-Associated Kaposi Sarcoma: Evidence of Symptom Palliation From Chemotherapy. Cancer (2010) 116(16):3969–77. doi: 10.1002/cncr.25362 PMC3157242 20564162

[31] Polizzotto MN Uldrick TS Wyvill KM Aleman K Peer CJ Bevans M. Pomalidomide for Symptomatic Kaposi's Sarcoma in People With and Without HIV Infection: A Phase I/II Study. J Clin Oncol (2016) 34(34):4125–31. doi: 10.1200/jco.2016.69.3812 PMC5477825 27863194

[32] Sekinaka Y Mitsuiki N Imai K Yabe M Yabe H Mitsui-Sekinaka K. Common Variable Immunodeficiency Caused by FANC Mutations. J Clin Immunol (2017) 37(5):434–44. doi: 10.1007/s10875-017-0396-4 28493158

[33] Tangye SG Al-Herz W Bousfiha A Chatila T Cunningham-Rundles C Etzioni A. Human Inborn Errors of Immunity: 2019 Update on the Classification From the International Union of Immunological Societies Expert Committee. J Clin Immunol (2020) 40(1):24–64. doi: 10.1007/s10875-019-00737-x 31953710 PMC7082301

